# Post-traumatic stress disorder and alcohol misuse: comorbidity in UK military personnel

**DOI:** 10.1007/s00127-016-1177-8

**Published:** 2016-02-10

**Authors:** M. Head, L. Goodwin, F. Debell, N. Greenberg, S. Wessely, N. T. Fear

**Affiliations:** King’s Centre for Military Health Research and the Academic Department for Military Mental Health, King’s College London, Cutcombe Road, London, SE5 9RJ UK

**Keywords:** Post-traumatic stress disorder, Alcohol, Comorbidity, Military

## Abstract

**Aims:**

To determine the prevalence of comorbid probable post-traumatic stress disorder and alcohol misuse in a UK military cohort study and to determine the level of co-occurrence between these disorders; further aims were to investigate the association between alcohol misuse and the different PTSD symptom clusters, and to assess what factors are associated with probable PTSD in participants with alcohol misuse.

**Methods:**

Data from 9984 participants of Phase 2 of the health and well-being survey of serving and ex-serving members of the UK Armed Forces were assessed for probable PTSD and alcohol misuse using the PTSD checklist (PCL-C) and the alcohol use disorders identification test (AUDIT), respectively.

**Results:**

1.8 % [95 % confidence interval (CI) 1.5–2.1] of the sample met the criteria for both PTSD and alcohol misuse. All three symptom clusters of PTSD were significantly associated with alcohol misuse, with similar odds ranging from 2.46 to 2.85. Factors associated with probable PTSD in individuals reporting alcohol misuse were age [ages 30–34 (years): OR 2.51, 95 % CI 1.15–5.49; ages 40–44 years: OR 2.77, 95 % CI 1.18–6.47], officer rank (OR 0.36, 95 % CI 0.16–0.85), being in a combat role in parent unit (OR 1.99, 95 % CI 1.20–3.31) and common mental disorder (CMD) (OR 21.56, 95 % CI 12.00–38.74).

**Conclusions:**

This study provides strong evidence that PTSD and alcohol misuse are often co-occurring. CMD was highly associated with probable PTSD in individuals with alcohol misuse.

## Introduction

Posttraumatic stress disorder (PTSD) and alcohol misuse are both potentially debilitating disorders, and the association between the two is well established [1–3]. Hazardous alcohol use or dependence is the most common comorbidity in males with PTSD [2], and the estimated prevalence of alcohol use disorders in individuals with PTSD is higher than that of the general population [4]. The association between PTSD and alcohol misuse has been evidenced across a number of countries [5–8]. A UK study found that 38.5 % of individuals with a substance use disorder (including alcohol) met DSM-IV criteria for current PTSD [9]. Results from the US National Comorbidity Survey (NCS) report a prevalence of PTSD of 10.3 % in men and 26.2 % in women who have alcohol dependence/misuse in the US general population, while the risk of developing alcohol dependence in those with previous PTSD was 2.67 and 3.37 times greater in men and women, respectively [10]. In the more recent NCS-replication, PTSD was associated with a higher risk (approximately four times the odds) of both alcohol dependence and alcohol abuse [11]. There is evidence for this comorbidity in studies of military personnel [3, 12, 13]. Alcohol misuse and PTSD also share common risk factors, for example combat exposure [14, 15], childhood adversity [16, 17] and common mental disorder [18, 19].

Whilst the association between PTSD and alcohol misuse has been established, the direction of causation is unknown. Several studies suggest that the comorbidity between alcohol misuse and PTSD represents an attempt at self-medication to alleviate the severity of PTSD-related symptoms [20–22], and that the development of new alcohol disorders in military personnel is uniquely predicted by higher levels of PTSD symptom severity [23]. It has also been found that substance-misuse patients with PTSD relapse more often and more quickly than those without comorbid PTSD [24, 25] and it has been suggested that such relapses are a response to resurgent PTSD symptoms [26]. Alternatively, alcohol misuse could be a risk factor for the development of PTSD. Davidson et al. found that alcohol misuse preceded PTSD by 3.1 years in a sample of US Vietnam veterans [27], and alcohol misuse may be associated with an increased rate of traumatisation due to the association between alcohol use and accidents and violence [28].

There is also evidence that particular PTSD symptoms are more strongly related to alcohol misuse than others; several studies suggest that avoidance and hyperarousal symptoms are more strongly associated with substance misuse in general [29], and specifically with alcohol misuse [30–33]. Another recent study, of Iraq and Afghanistan veterans, found that emotional-numbing symptoms of PTSD (a subset of the avoidance symptoms) were independently related to hazardous drinking in female veterans [34].

Several other studies have focussed on military veterans, with findings suggesting that US military personnel are at a higher risk of developing both alcohol misuse [35] and PTSD [36] than the general population. Studies from the UK also show higher rates of alcohol misuse in the military [37], but suggest that the prevalence of PTSD is similar to that in the general population [38, 39]. Whilst UK military studies have shown a strong association between PTSD and alcohol [18], the current study offers the opportunity to explore this relationship further. This study aims (1) to assess the comorbidity of PTSD and alcohol misuse in UK military personnel, (2) to determine the association of alcohol misuse with the three symptom clusters of PTSD, and (3) to examine what factors are associated with PTSD in those individuals who meet the criteria for alcohol misuse after accounting for current common mental disorders.

## Method

### Study design and participants

This study uses data gathered as part of Phase 2 of the health and well-being survey of serving and ex-serving members of the UK Armed Forces [38, 40]. This study collected data via self-complete questionnaires. Phase 1 of the survey was carried out between 2004 and 2006 [40], assessing the physical and mental health of the UK Armed Forces, comprising two samples. The first, termed the TELIC sample (the codename for the 2003–2009 UK military presence in Iraq), included personnel who deployed to Iraq between January 18, 2003, and April 28, 2003, and consisted of approximately 10 % of the fighting force deployed at that time. The second group, termed the Era sample, comprised individuals who were in the military during the same period, but were not at that time deployed to Iraq. In total, 10,272 participants completed the Phase 1 questionnaire (response rate 59 %).

**Fig. 1:**
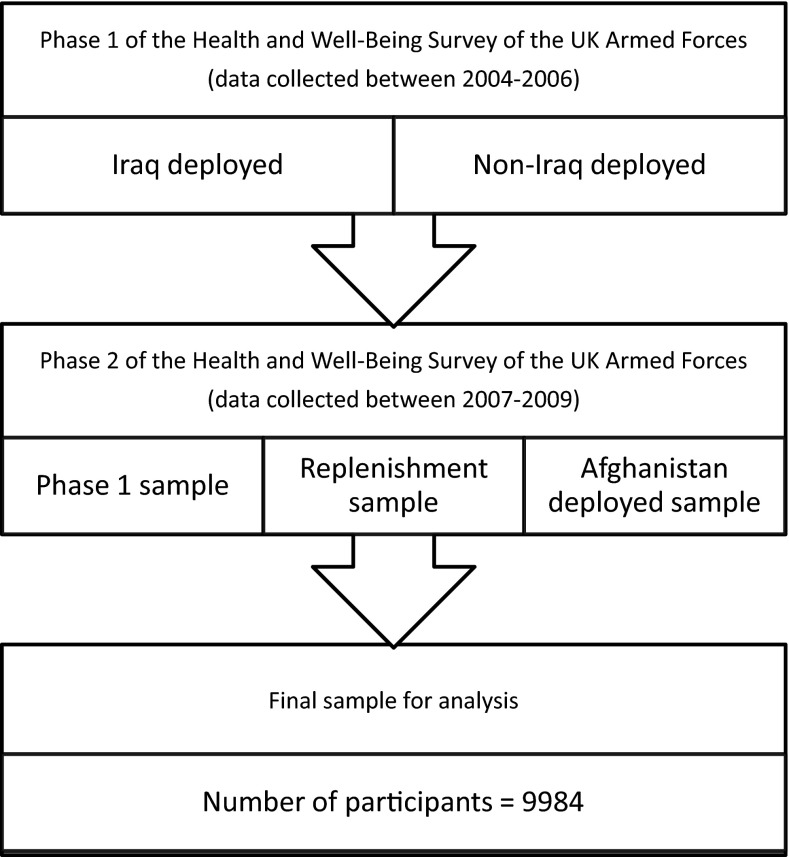
Flow diagram of study design and study participants

Phase 2 of the survey used a modified version of the Phase 1 questionnaire, with data collection occurring between 2007 and 2009. Participants from Phase 1 were re-contacted, with 6429 individuals (response rate 68.4 %) completing the Phase 2 questionnaire (now termed the follow-up sample). Two new samples were also included. The first was a random sample of personnel deployed to Afghanistan on operation HERRICK (the UK military codename for operations in Afghanistan) at any time between April 2006 and April 2007. 896 individuals (response rate 50.1 %) in this sample completed the Phase 2 questionnaire. The second new sample, termed the replenishment sample, included individuals who had joined the military since recruitment for Phase 1 in 2003, and had the opportunity to deploy on either TELIC or HERRICK during the study period. This sample was randomly drawn from those who joined the trained strength between April 2003 and April 2007, and 2665 individuals (response rate 40.2 %) completed the questionnaire. In total, 9990 individuals completed the Phase 2 questionnaire (overall response rate 56.0 %). Of these, six were later removed as they were found to be ineligible, resulting in 9984 individuals in the final sample (Fig. 1).

The socio-demographic characteristics of the Phase 2 sample were compared with the composition of the UK military (at April 2007), to ensure the sample was representative [38]. The distribution of sex, age, engagement type and rank was similar between groups, with service being the only exception; the sample included proportionally more army personnel than the UK military as a whole in 2007 (67 and 56 % respectively).

All participants were identified by Defence Analytical Services and Advice (DASA), UK Ministry of Defence (MoD), and ethical approval was provided by the King’s College Hospital local research ethics committee, and the MoD Research Ethics Committee (MODREC).

### Measures

To assess alcohol misuse, the Phase 2 questionnaire utilised the 10-item World Health Organization (WHO) alcohol use disorders identification test (AUDIT) [41]. The AUDIT assesses levels of alcohol consumption, alcohol dependence and negative consequences of alcohol abuse, in the previous 12 months. For the purposes of this study, participants were classified as reporting ‘alcohol misuse’ if they scored 16 or above. This cut-off is higher than that commonly used in the general population, and is defined by the WHO as ‘hazardous use that is also harmful to health’ [41].

For the assessment of PTSD symptoms, the Phase 2 questionnaire used the National Centre for PTSD Checklist—Civilian version (PCL-C) [42]. This is a 17-item questionnaire, and a cut-off of 50 or more was used to define caseness (‘probable PTSD’). Each question is rated on a scale from 1 to 5 depending on how often the individual has ‘been bothered by these problems’ in the past month, with 1 being ‘not at all’ and 5 being ‘extremely’.

The PCL-C can be divided into three PTSD symptom clusters [43], with 5 of the 17 questions assessing ‘re-experiencing’, 7 ‘avoidance’, and 5 ‘hyperarousal’ symptoms, corresponding to DSM-IV diagnostic criteria [44]. A rating of 4 or above (indicating the participant has been moderately bothered by that problem in the past month) was considered a positive response for that symptom being present. In order to determine caseness for the three symptom clusters, the following criteria were used: the ‘re-experiencing’ cluster required 1 or more positive symptom responses, the ‘avoidance’ cluster 3 or more positive symptom responses and the ‘hyperarousal’ cluster 2 or more positive symptom responses [43].

Symptoms of common mental disorder (CMD) including somatic symptoms, anxiety/insomnia, social dysfunction and depression were measured using the General Health Questionnaire 12 (GHQ-12) [45]. This assessed symptoms of CMD in ‘the last few weeks’, with caseness defined as a score of 4 or above (out of a possible 12) [46].

Two measures were used to assess childhood adversity [47], which were adapted from the Adverse Childhood Exposure study scale [48]. The first measured childhood family relationship adversity, by means of four positive items that were negatively scored (e.g., “I came from a close family”), and four negative items (e.g., “I used to be hit/hurt by a parent or caregiver regularly”) [47]. The scores for these eight items were summed to form a cumulative measure, and analysed as 0, 1 or 2+ adversities. The second measure assessed childhood antisocial behaviour, and was deemed positive if the participant a) answered ‘true’ to “I used to get into physical fights at school”, and b) answered true to one of the following: “I often used to play truant at school”; “I was suspended or expelled from school”; or “I did things that should have got me (or did get me) into trouble with the police” [49].

Age at Phase 2 questionnaire completion (in years), sex and marital status (single; married/cohabiting/in a relationship; separated/widowed/divorced) were assessed, as well as rank (commissioned officers; non-commissioned officers and other ranks), engagement type (regular or reservist), most recent deployment (Iraq; Afghanistan; no deployment) and whether the individual deployed in a combat role.

### Data analyses

#### Prevalence and comorbidity

The prevalence of probable PTSD and alcohol misuse were determined, and individuals meeting the criteria for both disorders were classified as comorbid.

#### Alcohol misuse and probable PTSD/PTSD symptom clusters

Logistic regression was used to determine the association between alcohol misuse and probable PTSD, as well as between alcohol misuse and the three PTSD symptom clusters. Unadjusted and adjusted odds ratios (ORs) [adjusted for socio-demographic and military factors—sex, age, marital status, rank, last deployment and engagement type (regular/reservist)] were calculated.

#### Factors associated with PTSD in individuals reporting alcohol misuse

Logistic regression was performed to determine the association between various socio-demographic/military factors (sex, age, marital status, military status, rank, last deployment, combat role, childhood adversity and common mental disorder) and probable PTSD within the alcohol misuse group. Unadjusted and adjusted ORs (and 95 % CIs) were calculated.

Although we have looked at the prevalence of PTSD in those scoring positively for alcohol misuse, the low overall prevalence of participants scoring positive for probable PTSD prevented us from having the statistical power to investigate what factors were associated with alcohol misuse in the PTSD group.

All analyses were performed using the statistical software programme STATA (version 11.0) [50]. All reported frequencies are unweighted, but percentages, odds ratios (ORs) and 95 % confidence intervals (CIs) are weighted to account for sampling fractions and probability of response [38]. Statistical significance was defined as *p* < 0.05.

## Results

### Sample characteristics

The majority of participants were male, in a relationship, under the age of 40 years, regulars of non-commissioned officer (NCO) or other rank, and more than half reported no previous deployment to either Iraq or Afghanistan (Table 1). A quarter of the sample reported that they were in a combat role in their parent unit. More than half of the sample reported at least one family relationship adversity in childhood and 17 % met the criteria for childhood antisocial behaviour. A fifth of the sample met the criteria for a CMD and 4 % met criteria for probable PTSD.

**Table 1:** Descriptive statistics for full sample at Phase 2 (*n* = 9984), and alcohol misuse group (*n* = 1323)

Variable	A	B
	Descriptive statistics for full Phase 2 sample (*n* = 9984)	Descriptive statistics for alcohol misuse group (*n* = 1323)
	*n* (column %)	*n* (column %)
Sex		
Male	8799 (89.7 %)	1230 (93.9 %)
Female	1185 (10.3 %)	93 (6.1 %)
Age at questionnaire completion (years)		
18–24	1684 (13.1 %)	416 (25.2 %)
25–29	1981 (19.9 %)	356 (29.2 %)
30–34	1628 (16.5 %)	187 (15.8 %)
35–39	1753 (19.0 %)	176 (14.0 %)
40–44	1461 (15.8 %)	115 (9.6 %)
45+	1477 (15.7 %)	73 (6.1 %)
Marital status		
In relationship	7581 (78.5 %)	810 (64.1 %)
Single	1714 (14.5 %)	405 (26.7 %)
Separated/widowed/divorced	639 (7.0 %)	100 (9.2 %)
Military status		
Regular	8274 (89.2 %)	1181 (93.5 %)
Reservist	1710 (10.8 %)	142 (6.5 %)
Rank		
NCOs/other ranks	7772 (80.3 %)	1172 (90.2 %)
Officers	2212 (19.7 %)	151 (9.8 %)
Last deployment		
No deployment	4999 (54.0 %)	521 (43.2 %)
Iraq (TELIC)	2679 (27.7 %)	437 (34.6 %)
Afghanistan (HERRICK)	2306 (18.3 %)	365 (22.2 %)
Combat role (in parent unit)		
Non-combat role	7527 (76.0 %)	872 (66.3 %)
Combat role	2340 (24.0 %)	434 (33.7 %)
Childhood family relationship adversity		
No adversity	4324 (44.2 %)	416 (32.2 %)
1 adversity	1920 (20.2 %)	261 (20.3 %)
2+ adversity	3408 (35.6 %)	593 (47.5 %)
Childhood antisocial behaviour		
No	8226 (82.9 %)	861 (65.1 %)
Yes	1593 (17.1 %)	439 (34.9 %)
Common mental disorder		
No	7943 (80.3 %)	844 (63.3 %)
Yes	1908 (19.7 %)	454 (36.7 %)
PTSD		
No	9502 (96.0 %)	1152 (86.4 %)
Yes	376 (4.0 %)	159 (13.6 %)

Weighted percentages (%) are presented

Numbers may not add up to total due to missing data

### Prevalence and comorbidity

376 participants [4.0 % (95 % CI 3.5–4.5)] were defined as having ‘probable PTSD’, with 1323 participants [13.0 % (95 % CI 12.3–13.8)] defined as reporting alcohol misuse. 1.8 % (95 % CI 1.5–2.1) of participants (*n* = 159) scored above threshold on both measures, and 84.8 % (95 % CI 83.4–85.6) did not score positive for either condition (*n* = 8251). In the group reporting probable PTSD, 44.9 % (95 % CI 0.4–0.5) had comorbid alcohol misuse, and within the group defined as reporting alcohol misuse, 13.6 % (95 % CI 0.1–0.2) also reported probable PTSD.

### Alcohol misuse and probable PTSD/PTSD-symptom clusters

Rates of probable PTSD were assessed in those who scored positive for alcohol misuse, and those who did not. Those scoring positive for alcohol misuse showed an approximate threefold increase in odds of having probable PTSD, compared to those without alcohol misuse (Table 2).

**Table 2:** Associations between alcohol misuse and PTSD/PTSD symptom clusters (odds ratios, adjusted odds ratios and 95 % confidence intervals)

	PTSD (*n* = 9772)			Re-experiencing (*n* = 9884)			Avoidance (*n* = 9879)			Hyperarousal (*n* = 9880)		
	*n* with PTSD (%)	OR (95 % CI)	Adjusted OR (95 % CI)	*n* with re-experiencing (%)	OR (95 % CI)	Adjusted OR (95 % CI)	*n* with avoidance (%)	OR (95 % CI)	Adjusted OR (95 % CI)	*n* with hyperarousal (%)	OR (95 % CI)	Adjusted OR (95 % CI)
Alcohol misuse												
No	210 (2.5 %)	1.00	1.00	1137 (13.0 %)	1.00	1.00	636 (7.6 %)	1.00	1.00	1154 (13.7 %)	1.00	1.00
Yes	159 (13.6 %)	6.14* (4.76–7.92)	3.53* (2.57–4.85)	436 (35.7 %)	3.73* (3.20–4.36)	2.46* (2.03–2.98)	309 (26.1 %)	4.28* (3.58–5.12)	2.80* (2.21–3.56)	484 (40.3 %)	4.27* (3.67–4.97)	2.85* (2.34–3.47)

ORs are adjusted for age, sex, marital status, military status, rank, last deployment, combat role, childhood family relationship adversity, childhood antisocial behaviour and CMD

Weighted percentages (%), odds ratios (OR) and 95 % confidence intervals (95 % CI) are presented

* = *p* < 0.05

All three PTSD symptom clusters were significantly associated with alcohol misuse compared to those without alcohol misuse, with a two- to threefold increase in odds for each cluster (Table 2).

### Factors associated with PTSD in the alcohol misuse group

The association between probable PTSD and various socio-demographic and military factors were examined in the group meeting the criteria for alcohol misuse. Individuals aged 30–34 years, and those aged 40–44 years, showed an approximate two- to threefold increase in odds for probable PTSD (compared to the reference category of participants aged 18–24 years) (Table 3). Officers had reduced odds of PTSD compared to non-commissioned officers and other ranks. Participants who were in a combat role in their parent unit showed a twofold increase in odds compared to participants in a non-combat role. The most strongly associated factor was CMD, with individuals scoring positive for CMD showing a 20-fold increase in odds for probable PTSD compared to those who did not. This may to a large extent be explained by the strong association between PTSD and CMD (results available from authors).

**Table 3:** Descriptive statistics and factors associated with probable PTSD in those meeting the criteria for alcohol misuse (*n* = 1323)

Covariate	Individuals scoring positive for probable PTSD in the alcohol misuse group (weighted row %, *n* = 159)	Unadjusted model, OR (95 % CI)	Adjusted model 1, OR (95 % CI)	Adjusted model 2, OR (95 % CI)
Sex				
Male	147 (13.4 %)	1.00	1.00	1.00
Female	12 (16.9 %)	1.31 (0.61–2.80)	1.86 (0.82–4.24)	1.22 (0.50–2.96)
Age at completion of survey (years)				
18–24	39 (10.7 %)	1.00	1.00	1.00
25–29	41 (13.3 %)	1.28 (0.74–2.22)	1.33 (0.71–2.51)	1.59 (0.81–3.14)
30–34	33 (20.9 %)	2.20 (1.23–3.93)*	2.28 (1.13–4.58)*	2.51 (1.15–5.49)*
35–39	15 (7.4 %)	0.66 (0.31–1.41)	0.72 (0.30–1.72)	0.80 (0.31–2.07)
40–44	22 (19.1 %)	1.98 (1.01–3.85)*	2.31 (1.06–5.06)*	2.77 (1.18–6.47)*
45+	9 (14.1 %)	1.37 (0.58–3.24)	1.97 (0.71–5.51)	2.95 (0.99–8.75)
Marital status				
In relationship	96 (13.3 %)	1.00	1.00	1.00
Single	39 (11.0 %)	0.80 (0.50–1.28)	0.80 (0.47–1.36)	0.78 (0.44–1.39)
Separated/widowed/divorced	23 (23.7 %)	2.02 (1.11–3.66)*	2.00 (1.07–3.74)*	1.34 (0.67–2.67)
Military status				
Regular	142 (13.5 %)	1.00	1.00	1.00
Reservist	17 (15.2 %)	1.14 (0.59–2.20)	1.16 (0.55–2.44)	0.92 (0.40–2.10)
Rank				
NCOs/other ranks	150 (14.3 %)	1.00	1.00	1.00
Officers	9 (7.9 %)	0.51 (0.23–1.12)	0.47 (0.21–1.07)	0.36 (0.16–0.85)*
Last deployment				
No deployment	65 (14.2 %)	1.00	1.00	1.00
Iraq (TELIC)	51 (13.6 %)	0.95 (0.61–1.51)	0.96 (0.58–1.57)	0.88 (0.51–1.52)
Afghanistan (HERRICK)	43 (12.6 %)	0.87 (0.54–1.41)	0.85 (0.49–1.48)	1.06 (0.59–1.93)
Combat role (in parent unit)				
Non-combat role	96 (12.0 %)	1.00	1.00	1.00
Combat Role	59 (16.1 %)	1.40 (0.93–2.11)	1.58 (0.99–2.52)	1.99 (1.20–3.31)*
Childhood family relationship adversity				
No adversity	45 (12.5 %)	1.00	1.00	1.00
1 adversity	33 (15.7 %)	1.30 (0.74–2.28)	1.36 (0.77–2.41)	1.52 (0.82–2.82)
2 + adversity	74 (13.9 %)	1.13 (0.71–1.79)	0.93 (0.56–1.53)	0.79 (0.45–1.38)
Childhood antisocial behaviour				
No	87 (12.4 %)	1.00	1.00	1.00
Yes	71 (16.5 %)	1.40 (0.94–2.08)	1.43 (0.91–2.23)	1.36 (0.83–2.20)
Common mental disorder				
No	20 (2.5 %)	1.00	1.00	1.00
Yes	139 (33.4 %)	19.9 (11.24–35.11)*	21.56 (12.0–38.74)*	21.56 (12.00–38.74)*

* *p* < 0.05

Model 1 is adjusted for age, sex, marital status, military status, rank, last deployment, combat role, childhood family relationship adversity, and childhood antisocial behaviour

Model 2 is adjusted for age, sex, marital status, military status, rank, last deployment, combat role, childhood family relationship adversity, childhood antisocial behaviour and CMD

Weighted percentages, ORs and 95 % CI are presented

Numbers may not add up to total due to missing data

## Discussion

This study has established that the prevalence of comorbid PTSD and alcohol misuse in the UK military is 1.8 %, with 44.9 % of those with probable PTSD reporting alcohol misuse, and 13.6 % of those with alcohol misuse also meeting the criteria for PTSD. We found that all three symptom clusters of PTSD were reported significantly more often in those with alcohol misuse than those without. In those reporting alcohol misuse, we found a strong significant association between PTSD and CMD, as well as significant associations with age, rank and combat role.

This study found that the prevalence of probable PTSD within the group scoring positive for alcohol misuse was 13.6 %. This is slightly less than previous studies have found, with the rate of PTSD in those with alcohol misuse commonly reported as 15–30 % [51–53] and sometimes greater than 50 % [54]. However, 13.6 % is still higher than the prevalence of PTSD in the general military population (4 %), suggesting a strong association between the two disorders.

All three PTSD symptom clusters were strongly associated with alcohol misuse, and this study does not support previous findings that the ‘avoidance’ cluster of symptoms was more strongly associated with alcohol misuse [29–33, 55]. Our findings are in line with those of Johnson [56] and Taft [57], who found that all three symptom clusters were similarly (and significantly) associated with alcohol misuse.

In those individuals who met the criteria for alcohol misuse, there was a statistically significant association between combat role and PTSD. This fits with other military studies which have found higher rates of PTSD in those deployed in a combat role [58]. The strongest association, however, was found between CMD and PTSD (in those with alcohol misuse). This may be a result of the well-established associations between substance misuse, PTSD and depression [59–61]. A number of recent studies have looked at the interplay between these factors. A recent meta analysis found that 52 % of those with PTSD had comorbid major depressive disorder [19]. Khoury et al. found that PTSD symptom scores in a lifelong alcohol-dependent group were no longer significantly different from non-dependents once depressive symptoms were controlled for [33]. Similarly, other studies have found that the PTSD risk in those with alcohol misuse is no longer significant when adjusted for depression risk [61], and that depression is significantly associated with PTSD and alcohol misuse comorbidity [62].

The present study found no significant association between childhood adversity and PTSD in those reporting alcohol misuse. This is counter to the findings of several studies [63, 64], including those using samples drawn from both the UK [47] and US military populations [65, 66]. There may be other childhood or pre-enlistment risk factors for PTSD which we were not able to measure in the current study and there is potential for an interaction between childhood adversity and combat exposure, with a greater PTSD risk in those exposed to combat who had already experienced traumatic events in childhood [66].

### Study strengths and limitations

Most studies looking at PTSD and alcohol misuse in military populations have focussed on the US Armed Forces, and by drawing data from the UK military, this study has broadened the scope of research in this field. This study also investigated the three symptom clusters of PTSD separately, a distinction not made in all research. Furthermore, although all data were gathered using a self-report method, the measures included in the questionnaire (e.g., AUDIT, PCL-C) have well-established reliability and validity.

There are various aspects of the association between alcohol abuse and PTSD that are not addressed in this study. Although we have looked at the prevalence of PTSD in those scoring positively for alcohol misuse, the low overall prevalence of participants scoring positive for probable PTSD prevented us from having the statistical power to investigate what factors were associated with alcohol misuse in the PTSD group. Also, some studies separate the ‘avoidance’ cluster of PTSD symptoms into two groups—‘avoidance’ symptoms and ‘numbing’ symptoms—and they suggest it is the ‘numbing’ symptoms that are more strongly associated with alcohol misuse [67, 68]. By not dividing the ‘avoidance’ cluster this association could potentially be missed. This distinction was not made in the present study, due to the low reported prevalence of PTSD in our sample. Further this study was based on the DSM-IV criteria for PTSD using the PCL. Recently with the introduction of the DSM-V criteria, the three symptom clusters (intrusive recollections, avoidant/numbing symptoms, and hyper-arousal symptoms) have been replaced by four symptom clusters (intrusion, avoidance, negative alterations in cognitions and mood, and alterations in arousal and reactivity). It will be essential that the revised criteria are explored in subsequent studies which use the PCL-5.

### Implications

Although the majority of military personnel do not report alcohol misuse or PTSD, this study has demonstrated that PTSD and alcohol misuse do co-occur with approximately 50 % of those with probable PTSD also reporting alcohol misuse. This has implications for clinicians who should ensure that individuals presenting with either condition are assessed for comorbidity. Therefore, treatment for PTSD should take the alcohol misuse into account rather than treating the conditions consecutively. This might help identify the cause of their initial presentation (e.g., heavy drinking to alleviate the symptoms of undiagnosed PTSD), and in turn lead to more effective treatment and counselling programs. The role of CMD is also important to consider as some individuals reported all three conditions, this level of co-morbidity is likely to impact on presentation and treatment priorities. However, these cross-sectional results do not clarify the direction of the association.

## Conclusion

The present study has demonstrated the co-occurrence of alcohol misuse and PTSD in the UK military and provides further evidence that other factors, most notably CMD, are also associated with this comorbidity.
